# QTL mapping identifies novel major loci for kernel row number-associated ear fasciation, ear prolificacy and tillering in maize (*Zea mays* L.)

**DOI:** 10.3389/fpls.2022.1017983

**Published:** 2023-01-10

**Authors:** Kai Li, Alberto Tassinari, Silvia Giuliani, Serena Rosignoli, Claude Urbany, Roberto Tuberosa, Silvio Salvi

**Affiliations:** ^1^ Department of Agricultural and Food Sciences (DISTAL), University of Bologna, Bologna, Italy; ^2^ KWS SAAT SE & Co. KGaA, Einbeck, Germany

**Keywords:** ear fasciation, ear prolificacy, maize, QTL mapping, tillering, yield components

## Abstract

Maize ear fasciation originates from excessive or abnormal proliferation of the ear meristem and usually manifests as flattened multiple-tipped ear and/or disordered kernel arrangement. Ear prolificacy expresses as multiple ears per plant or per node. Both ear fasciation and prolificacy can affect grain yield. The genetic control of the two traits was studied using two recombinant inbred line populations (B73 × Lo1016 and Lo964 × Lo1016) with Lo1016 and Lo964 as donors of ear fasciation and prolificacy, respectively. Ear fasciation-related traits, number of kernel rows (KRN), ear prolificacy and number of tillers were phenotyped in multi-year field experiments. Ear fasciation traits and KRN showed relatively high heritability (*h* ^2^ > 0.5) except ratio of ear diameters. For all ear fasciation-related traits, fasciation level positively correlated with KRN (0.30 ≤ *r* ≤ 0.68). Prolificacy and tillering were not correlated and their *h* ^2^ ranged from 0.41 to 0.78. QTL mapping identified four QTLs for ear fasciation, on chromosomes 1 (two QTLs), 5 and 7, the latter two overlapping with QTLs for number of kernel rows. Notably, at these QTLs, the Lo1016 alleles increased both ear fasciation and KRN across populations, thus showing potential breeding applicability. Four and five non-overlapping QTLs were mapped for ear prolificacy and tillering, respectively. Two ear fasciation QTLs, *qFas1.2* and *qFas7*, overlapped with fasciation QTLs mapped in other studies and spanned *compact plant2* and *ramosa1* candidate genes. Our study identified novel ear fasciation loci and alleles positively affecting grain yield components, and ear prolificacy and tillering loci which are unexpectedly still segregating in elite maize materials, contributing useful information for genomics-assisted breeding programs.

## 1 Introduction

Fasciation is a deviational proliferation of cells and tissues eventually manifesting as widened and flattened organs (most commonly stems or inflorescences) that has been reported in more than a hundred plant families, including trees, shrubs and grasses (White, 1948; Iliev and Kitin, 2011). Ontogenetically, fasciation has been interpreted as (i) an excrescence or fusion of organs due to deviations from normal meristematic processes or crowding of buds, or (ii) a transformation of a single growing meristematic point into a line, this sometime called ‘true fasciation’ (Clark et al., 1993; Iliev and Kitin, 2011). Either way, mutations at genes involved in the maintenance and functions of the shoot apical meristem (SAM) and the early generative inflorescence meristem play a major role in fasciation, along with environmental factors (Somssich et al., 2016; Wu et al., 2018; Ortez et al., 2022).

Based on studies first carried out in *Arabidopsis*, SAM homeostasis was shown to be controlled by the *CLAVATA3* (*CLV3*) - *WUSCHEL* (*WUS*) feedback signaling pathway (Brand et al., 2000; Schoof et al., 2000; Somssich et al., 2016). WUS is a stem cell-promoting homeodomain transcription factor, whereas CLV3 is a differentiation-promoting peptide that belongs to the CLAVATA3/EMBRYOSURROUNDING REGION (ESR) CLE peptide family (Opsahl-Ferstad et al., 1997; Trotochaud et al., 1999). While WUS activates the expression of *CLV3*, *WUS* expression is repressed by CLV3 through its binding to a number of leucine-rich repeat receptor-like proteins (including CLV1 and CLV2), causing the decline of stem cell proliferation, and a corresponding decrease of CLV3 production (Brand et al., 2000; Schoof et al., 2000). This feedback mechanism keeps meristem size under control and appears to be largely conserved in maize. For instance, maize *CLV3/EMBRYO-SURROUNDING REGION7* (*ZmCLE7*) is a *CLV3* ortholog; *THICK TASSEL DWARF1* (*TD1*) and *FASCIATED EAR2* (*FEA2*) encode receptor-like proteins related with CLV1 and CLV2, and regulates the size of the ear IM and SAM growth; *ZmWUS1* seems to be a direct *WUS* ortholog and is expressed in the late vegetative SAM (Wu et al., 2018; Kitagawa and Jackson, 2019; Kim et al., 2022). Genes affecting meristem size and involved in ear fasciation and acting outside the CLV3-WUS loop have also been described. For instance, *ZmFEA4* encodes a basic leucine zipper domain (bZIP) transcription factor that is expressed in the shoot meristem peripheral zone and is likely involved in cell transiting from SAM to organ primordium (Kitagawa and Jackson, 2019); the SQUAMOSA PROMOTER BINDING (SBP)-box transcription factors *unbranched2* (*UB2*) and *unbranched3* (*UB3*) are expressed in the initiating leaf primordia and the base of the SAM and control lateral organs initiation (Chuck et al., 2014). Finally, ear shape is also under the control of genes expressed later in development, at the inflorescence or even floret meristem levels, including *ramosa1* (*ra1*), *ra2* and *ra3* (Vollbrecht et al., 2005; Kellogg, 2022), or *growth regulating factor-interacting factor1* (*gif1*. Zhang et al., 2018), which were shown to control inflorescence (tassel and ear) branching and, when mutated, to produce multiple-tip or branched ears.

Fasciated mutants can be of interest in plant breeding programs for their ornamental characteristics or because their abnormal development may favorably affect yield components such as fruit and/or seed size and number. Indeed, fasciated tomato mutants showed increased number of flowers, fruits, fruit locules and fruit size (Tanksley, 2004; Iliev and Kitin, 2011; Xu et al., 2015). Similarly, mutations in *CLV* genes in *Brassica* resulted in higher number of locules in fruits, leading to higher yield (Fan et al., 2014; Xiao et al., 2018). In maize, ear fasciation has been suggested as a potential target for increasing ear size and/or number of kernels per ear under the expectation that a larger SAM should lead to a wider ear meristem eventually harboring more spikelet pair meristems and thus kernel rows (Otegui and Bonhomme, 1998; Somssich et al., 2016; Kim et al., 2022; Li et al., 2022). However, accumulating evidences indicate that strong ear fasciation alleles do not improve productivity because they usually induce a shorter, stunted ear; instead, weaker fasciation alleles show more potential (Bommert et al., 2013b; Je et al., 2016; Kim et al., 2022; Ortez et al., 2022). In line with this evidence, quantitative genetic variation for ear fasciation was analyzed using QTL mapping (Mendes-Moreira et al., 2015) and, in a different study, a QTL for kernel row number (KRN) was shown to correspond to *ZmFEA2* (Bommert et al., 2013b). A different QTL on the same chromosome explained 12% of KRN variance, was cloned and shown to correspond to a cis-regulatory element of *UB3* (Chuck et al., 2014; Du et al., 2020).

In maize, prolificacy is a general term indicating the presence of multiple ears in a plant (Ortez et al., 2022). Prolificacy can be classified as three types: multi-node prolificacy (i.e., multiple ears growing at different nodes), multi-tiller prolificacy (i.e., multiple stems growing from basal nodes) and single-node prolificacy [i.e., multiple ears growing at the same nodes, also known as ‘multi-ears’, ‘bouquet ears’ or ‘shank ears’ (Ortez et al., 2022)]. In single-node prolificacy, the presence of multiple ears is the result of multiple axillary meristems located on the same ear shank, giving rise to additional ear inflorescences alongside the central one. The presence of a single major ear per plant *vs*. multiple ears is one the major contrasting difference between currently cultivated maize and its progenitor teosinte (Stitzer and Ross-Ibarra, 2018). Because of this, domesticated maize was referred as ‘not prolific’, while its progenitor teosinte as ‘prolific’ (Doebley et al., 1995; Prakash et al., 2019; Yang et al., 2019). Although most of the modern maize hybrid cultivars cultivated in the high-dense stands in temperate environment develop only one ear, the potential presence of multiple ears per plant has physiological and breeding implications. For instance, maize hybrid cultivars with some level of plasticity to develop tillers and multiple ears per plant may turn out advantageous in semi-arid regions with high inter-annual variation of summer rainfall, where they are cultivated at low plant population densities (i.e., less than 4 plants m^-2^. Rotili et al., 2022). Additionally, ear prolificacy is considered a positive feature in ‘baby corn’ specialty maize cultivars whose unfertilized ears are consumed in salads, soups, fried snacks and other ways (Prakash et al., 2019). The genetic control of multiple ears per plant is complex and received little attention so far. A major QTL for single-node prolificacy, *prol1.1*, was mapped on chr. 1 in a maize-teosinte BC_2_S_3_ population, at a chromosomal location that had previously been shown to influence domestication traits and shown to correspond to the expression regulatory region of *grassy tillers1* (*gt1*), encoding a homeodomain leucine zipper transcription factor (Wills et al., 2013). More recently, major multi-node prolificacy QTLs were mapped in different crosses (Prakash et al., 2021; Wang et al., 2021).

Ears and tillers both originate from axillary buds developing into shorter or longer shoots, therefore they are expected to share developmental mechanisms. This was indeed confirmed by the identification and cloning of genes such as *barren stalk1* (*ba1*), encoding a bHLH transcription factor orthologous to rice *LAX PANICLE1* (*LAX1.* Gallavotti et al., 2004) . Additionally, *ba1* mutants fail to initiate all vegetative and reproductive axillary meristems. BA1 levels are under the control of BARREN STALK FASTIGIATE1 (BAF1), a transcriptional regulator with an AT-hook DNA binding motif (Gallavotti et al., 2011). Mutant *baf1* plants fail to initiate axillary buds that are fated to become lateral ear shoots; as a result, *baf1* mutants are earless (Gallavotti et al., 2011).

This study investigated the genetic control of ear fasciation and ear prolificacy and their links with KRN and tillering, respectively, using QTL mapping. Mapping was carried out in two connected (ie. sharing one parental line) RIL populations, B73 × Lo1016 and Lo964 × Lo1016 analyzed as a joint population. Line Lo1016 was the genetic source of mild ear fasciation while Lo964 was the source of ear prolificacy.

## 2 Materials and methods

### 2.1 Plant materials and marker genotyping

Two recombinant inbred line (RIL) populations were developed as follows. The line Lo1016 was crossed with B73 and with Lo964 to create two F_1_s. Both Lo964 and Lo1016 are typical dent lines originally bred at the Bergamo breeding station (Italy), shown to be genetically related based on molecular marker analysis and classified as BSSS = Iowa Stiff Stalk Synthetic heterotic group (Losa et al., 2011). B73 is the maize reference inbred line (Schnable et al., 2009). Single seed descent method was utilized to reach F_7_ generation, after which each line was multiplied following standard procedures. The B73 × Lo1016 (B×L) and Lo964 × Lo1016 (L×L) populations eventually included 97 and 68 RILs, respectively. We will refer to the Joint Population (JP) as the assembly of the whole set of 165 (97 + 68) RILs.

### 2.2 Field experiment and phenotypic data collection

Field trials were carried out in Cadriano, near Bologna, Italy (44°33’02.5”N, 11°24’43.9”E) in 2017 and in Monselice, near Padova, Italy (45°12’42.4”N, 11°45’14.8”E) in 2018 and 2019. Field experiments were organized as a randomized complete block design with two replicates (one rep = one plot with 10 plants per RIL). Plots for each of the three parental lines were included in the experiment. Plot length was 2.5 m, distance between rows was 0.8 m and between plants was 0.25 m. Plots were overplanted by hand and thinned at the V7 growth stage to one plant per hill equivalent to 11 plants per plot, and with an overall investment of 5.5 plants per square meter. The field was managed following standard agronomic practices of the area.

Phenotyping for ear fasciation was carried out by collecting four traits, namely ‘ear ovality’ (OVA, defined after visual inspection of elliptic/ovality degree of cob cross-section, from 0 to 10, corresponding from perfect circle to extremely elliptic/flat cob cross sections, respectively; higher values indicated strong fasciation); ‘kernel row disorder’ (DIS, defined as a visual score from 0 = perfectly linearly arranged kernels on ears, to 10 = highly disordered arrangement; higher values indicated strong fasciation); ‘ear diameters ratio’ (DIA, defined as ratio of minimum diameter divided by maximum diameter, where the two diameters were measured mutually perpendicularly by a caliper at the middle of the ear; lower values corresponded to strong fasciation); ‘ear fasciation index’ (FAS, a visual score for ear fasciation scaled from 0 to 3, where 3 indicated a strongly fasciated ear). Visual scores per ear were given by three persons independently, and mean values were utilized as entries for subsequent analysis. Number of kernel rows (KRN) was collected by counting the number of kernel rows at mid-ear position, on the same ears subjected to phenotyping for fasciation. Plant architecture traits collected were number of tillers per plants (TIL) and proportion of plants per plot showing prolificacy (PROL), i.e., proportion of plants showing >1 ear at the top ear node. For all traits, three central contiguous plants per plot were considered.

**Figure 1:**
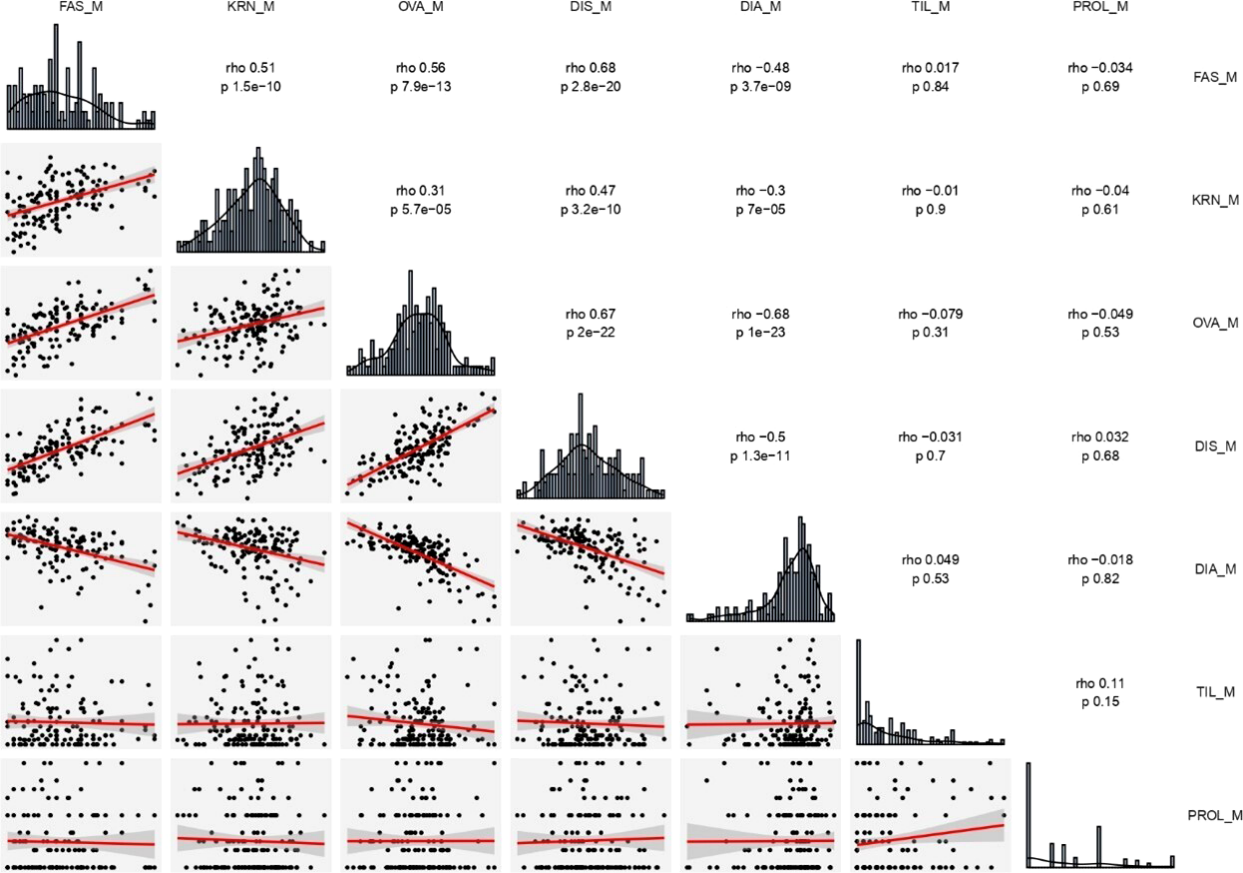
Distribution frequency histograms of, and correlation among all traits estimated on the two RILs (B×L and L×L) combined. The upper right part reports all correlation indexes (*rho*) and corresponding significant levels (*p*). The lower left part presents scatter plots and fitter curve (the red line inside) between two traits. The diagonal shows histogram charts of each trait. DIA (ear diameters rate), DIS (kernel row disorder), OVA (ear ovality), FAS (ear fasciation index), KRN (kernel row number), PROL (prolificacy), TIL (number of tillers), M, mean value.

Raw phenotypic data for all traits were modified by using the model Best Linear Unbiased Estimator (BLUES) in the R package “tidyverse” (Wickham et al., 2019). BLUES values were utilized for biometric, correlation and QTL analyses. Correlation analysis was carried out by the Spearman method which is little sensitive to deviation from normal distributions (Wickham et al., 2019). We utilized the common letter ‘*r*’ instead of the more appropriated ‘*rho’* for clarity in text. Trait distributions were normal or reached normality after square root transformation (Sokal and Rohlf, 2012), except for ear diameters rate, prolificacy and tillering (**Supplementary Table 1**). We used original data for visualization (**Figure 1** and **Supplementary Figures 1, 2**).

Analysis of variance (ANOVA) was run for six out of seven traits and for two (2018 and 2019, corresponding to experiments in Monselice, near Padua) out of three years. Specifically, ANOVA was not run for ear fasciation index since this phenotypic dataset was only available as mean value of two repetitions. Similarly, phenotypic data for year 2017 were not included in ANOVA because mean values (as mean of two repetitions) were available only. ANOVA output tables for six traits are provided in **Supplementary Table 2**. For each trait, ANOVA had three sources of variation, namely Year (2018 and 2019), Population (B×L, and L×L) and RIL (97 RILs for B×L and 68 RILs for L×L). RILs were treated as ‘Nested’ in Population. The final linear model utilized (in R) was Trait ~ Year * Population/RIL. Year was considered as random while Population and RIL factors were considered as fixed effect.

### 2.3 SNP genotyping, construction of linkage map and QTL mapping

B×L and L×L were genotyped using a high density 15-K SNP array (Rousselle et al., 2015) using a commercial service. Genomic DNA was prepared following standard protocols by a DNA extraction kit (NucleoSpin Plant II, Macherey Nagel, Duren, Germany) based on the manufacturer’s protocol. Marker alleles from Lo1016 were coded as 0 while alleles from Lo964 and B73 were coded as 2 and missing values as -1. Linkage map construction was obtained using QTL Icimapping (Meng et al., 2015) by first removing redundant markers using the procedure “BIN”, and then building linkage maps using “MAP”. Three linkage maps were constructed, one for each biparental cross RIL population, and one as joint population. For “BIN” function, markers whose missing rate was higher than 50% and distortion rate higher than 0.01 were deleted. For “MAP” function, the algorithm nnTwoOpt and SARF (sum of adjacent recombination fractions) for rippling were applied, and the window size was specified as 9. QTL mapping was carried out by QTL Icimapping using the Nested Association Mapping (NAM) functionality, treating the two subpopulations (B×L and L×L) as a single population (ie. Joint Population, JP). This was possible because the two subpopulations share on common parent (Lo1016), making it equivalent to a NAM design (Li et al., 2011). Under NAM functionality, QTL Icimapping applies ‘joint inclusive composite interval mapping’ (JICIM. Li et al., 2011). The JICM-based QTL analysis was proved to provide higher QTL detection power and mapping precision as compared to the analysis of the single biparental populations (Li et al., 2011). In parallel, we carried out QTL analysis on single subpopulations (using the options BIP and composite interval mapping in QTL Icimapping) for checking purposes. The scanning step was set as 0.1 cM in NAM and 1 cM in biparental populations, respectively. Probability of stepwise regression was set to 0.0001. The LOD threshold for declaring QTL significance was set as 3.3, 3.6 and 4.6, for B×L, L×L and JP, respectively, after permutation test (*P* ≤ 0.05 with 1,000 permutation). QTL additive effects were always computed by the formula 2a = (mean homozygous B73 – mean homozygous Lo1016) or 2a = (mean homozygous Lo964 – mean homozygous Lo1016), for the two RIL populations, respectively.

Additionally, QTL mapping for ear diameters rate, ear prolificacy and tillering (ie. traits showing deviation from normal distribution) was also carried out by Kruskal-Wallis (KW) test, known to be robust to deviation from distribution normality, as implemented in MapQTL6 (Van Ooijen, 2009). For these traits, QTL results obtained both with JICIM and KW tests are provided and compared in **Supplementary Table 3**. Since the two methods basically provided the same results, results from KW test will be no further discussed, with the exception of QTLs *qProl1* and *qProl4*, for which we provided footnotes in **Table 2**.

### 2.4 Screening candidate genes and variant calling

QTL confidence intervals from this study were projected on B73 reference genome (B73v5. Hufford et al., 2021) and included gene models that were considered for further investigations. Whole genome sequencing of the two lines Lo964 and Lo1016 was carried out with Illumina HiSeq PE150 at 20× of coverage. Reads were aligned to the B73v5 using BWA v.7.17 (Li and Durbin, 2009). Variants were called with BCFtools v. 1.10.2 (Li, 2011) and were filtered for a minimum reads depth of 10×, PHRED quality > 40 and a minimum DV/AD ratio of 0.8, where DP is the coverage depth at the variant position and AD is the allelic depth of the alternate allele. Variant effects were predicted with SNPEff v.3.0.7 (Cingolani et al., 2012) and among variants in the gene space, only high or moderate effects were considered. Additionally, alleles sequences of candidate genes were extracted for Lo964 and Lo1016 from their whole variant call format (vcf) files and the FASTA sequences were obtained with the command bcftools consensus. The 25 NAM founder sequences were downloaded from MaizeGDB (Portwood et al., 2019). The FASTA sequences were aligned using MUSCLE (Edgar, 2004) from MEGAX (Kumar et al., 2018). The coding sequences were obtained starting from the genomic sequence and the B73v5_Zm00001eb.1.gff3 annotation file downloaded from MaizeGDB, using GFFRead (Pertea and Pertea, 2020). The alignment images were elaborated with Jalview (Waterhouse et al., 2009). Finally, a review of published QTLs and genes in maize was carried out by searching major bibliographic databases using ‘ear fasciation’, ‘prolificacy’ or ‘tillering’ terms as keywords, and information on QTL and genes physical position, bin and type of mapping population was collected. Gene name formats and symbols followed the indications given at www.maizegdb.org/nomenclature.

## 3 Results

### 3.1 Trait biometrics, heritability and phenotypic correlations

**Figure 2:**
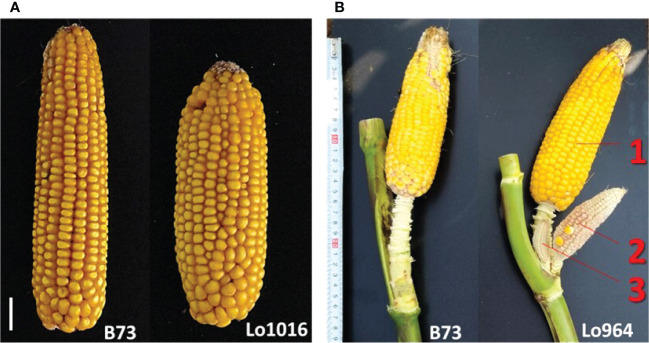
Target ear traits analyzed in this study. **(A)** Representative images of the ear-fasciation phenotype observed in Lo1016 (B73 is shown as comparison). White line, 1 cm. **(B)** Representative images of the ear prolificacy phenotype at top ear-bearing node as observed in Lo964 (B73 is shown as comparison). Numbers (1-3) indicate different ears at the same node.

This study confirmed that Lo1016 and Lo964 are characterized by ear fasciation and ear prolificacy, respectively (**Figure 2**, **Supplementary Figure 1**, **Table 1**). Specifically, Lo1016 showed the highest fasciation values when compared to Lo964 and B73 except kernel row disorder (Tab. 1. P < 0.01 for all comparisons for ear diameters rate, ear fasciation index and ear ovality/flatness, Tukey’s test). Alongside, Lo1016 also showed the highest KRN (19.75 *vs* 16.33 or 14.54, for B73 or Lo964, respectively, *P* < 0.01). Lo964 showed the highest ear prolificacy among the three parental lines (2.75 *vs* 0.0 or 0.25, for B73 or Lo1016, respectively, *P* < 0.01). In addition, Lo1016 was the only parental line developing tillers (ca. 1.5 tillers per plant). Values for additional plant architecture traits recorded in this study are reported in **Table 1**. ANOVA showed that ear diameters rate, kernel row disorder, KRN and prolificacy were strongly affected by ‘Year’ (*P* < 0.01) while ear ovality and number of tillers were only mildly affected (0.01 < *P* < 0.05) (**Supplementary Table 2**). ‘Population’ significantly affected all traits (*P* < 0.01) except number of tillers. No ‘Population-Year’ interactions were observed for any traits with the exception of prolificacy. For any trait, RIL (ie. lines) was a major source of variation, in line with the presence of segregating QTLs. Broad sense heritability (*h* ^2^) for ear-fasciation traits ranged from 0.13 for ear diameters rate in L×L to 0.95 for ear fasciation index in B×L. Ear prolificacy and TIL *h* ^2^ were relatively high (ranged between 0.41 and 0.78, **Table 1**).

**Table 1:** Descriptive statistics for ear and plant architecture traits in the two RIL populations B73 × Lo1016 (B×L) and Lo964 × Lo1016 (L×L).

	B73	Lo964	Lo1016	RIL population B×L		RIL population L×L	
Traits^†^	Mean ± sd	Mean ± sd	Mean ± sd	Min - Mean - Max	*h* ^2^	Min - Mean - Max	*h* ^2^
DIA	0.95 ± 0.04 (a)	0.95 ± 0.04 (a)	0.85 ± 0.11 (a)	0.86 - 0.94 - 0.98	0.45	0.88 - 0.95 - 0.98	0.13
DIS	7.17 ± 1.18 (a)	6.33 ± 1.89 (a)	7.18 ± 0.94 (a)	2.75 - 6.02 - 8.89	0.58	3 - 5.25 - 8.61	0.63
FAS	0.18 ± 0.39 (a)	0.50 ± 0.50 (a)	2.20 ± 0.75 (b)	0 - 1.38 - 3	0.95	0 - 0.79 - 2.92	0.94
KRN	16.33 ± 1.20 (a)	14.54 ± 1.03 (a)	19.75 ± 1.71 (b)	14.58 - 17.40 - 20.90	0.59	12.67 - 15.97 - 20.13	0.85
OVA	5.85 ± 1.03 (a)	4.92 ± 1.82 (a)	7.13 ± 1.17 (b)	4.1 - 6.02 - 8.11	0.50	3.69 - 5.60 - 8.11	0.55
PROL	0 ± 0 (a)	2.75 ± 2.36 (b)	0.25 ± 0.5 (a)	0 - 0.40 - 1	0.41	0 - 0.59 - 1	0.78
TIL	0 ± 0 (a)	0 ± 0 (a)	1.5 ± 2.38 (b)	0 - 1.22 - 5.75	0.62	0 - 1.24 - 5.25	0.68

Different letters (a and b) indicate statistical significance, Tukey’s test (*P* < 0.05).

^†^) DIA (ear diameters rate), DIS (kernel row disorder), OVA (ear ovality), FAS (ear fasciation index), KRN (kernel row number), PROL (prolificacy), TIL (number of tillers).

Positive transgressive segregation was observed for TIL only, with some RIL lines belonging to both populations that showed > 5 tillers per plant as compared to 1.5 tillers per plant on average in the high-tillering parent Lo1016. Negative transgressive segregation was observed for KRN, with RIL lines from L×L showing as few as 12.7 kernel rows as compared to 14.5 or 19.7 kernel rows recorded for Lo964 or Lo1016. Negative transgressive segregation was also observed for DIS in both RIL populations.

The four ear-fasciation-related traits (ear diameters rate, ear fasciation index, ear ovality, kernel disorder) resulted significantly correlated, with *r* ranging from |0.48| to |0.68| (*P* < 0.001, **Figure 1**, **Supplementary Figures 2, 3**), with ear diameters rate negatively correlated as expected (i.e., the smaller the rate, the stronger ear fasciation). Additionally, the same traits correlated with KRN. Among ear fasciation-related traits, ear fasciation index showed the highest correlation (*r* = 0.51) with KRN and ear diameters rate the lowest (*r* = −0.30, *P* < 0.001. **Figure 1**), again with ear diameters rate as the only trait negatively correlated with KRN. Overall, the correlation results suggested that variation for KRN and ear fasciation could partially be due to the same loci. Ear fasciation traits did not show correlation with ear prolificacy or tillering (**Figure 1**).

### 3.2 Linkage maps

The three linkage maps, namely B×L, L×L and JP included 1,186, 984 and 1,303 markers, and covered 1,819.52 cM, 2,504.5 cM and 1,661.0 cM, respectively (**Table 2** and **Supplementary Figure 4**). The different linkage maps covered well the maize genome with the unavoidable exception of those regions characterized by lack of markers polymorphism due to identity-by-descent between lines. In B×L, those regions were identified as the middle part of chr. 1 between PZE-101130395 (168,493,734) and PZE-101137700 (180,295,042), accounting for 3.8% of the chromosome, and the upper and lower parts of chr. 3, for a total of 88.7 Mb (37.6% of chr. 3). In L×L, almost the whole chr. 3 resulted monomorphic and thus uninformative for QTL mapping. Additionally, deficits of polymorphic markers resulted in long intervals between markers on the upper parts of chr. 4 and 7, accounting for 4.9 and 7.0% for each corresponding chromosome, respectively. Overall, 87.7 and 75.7% of the maize map was sufficiently covered by molecular markers in B×L and L×L, respectively.

**Table 2:** QTL results for ear-fasciation (and related traits), kernel row number, ear prolificacy and tillering as obtained by composite interval mapping using BLUES-modified phenotypic values, on single RIL populations (B×L and L×L) and by analysis of joint population (JP).

Trait type	QTL	Trait	Source^a^	Genetic^b^	Bin	Physical B73v4Gramene	LOD^c^	PVE^d^	LOD B×L^e^	LOD L×L^f^	Add B×L^g^	Add L×L^h^
Ear fasciation and KRN	*qFas1.1*	Ear diameter rate	B×L	chr1:18	1.01	1:4,727,090.5,522,697	3.97	13.35			0.01	
	*qFas1.2*	Fasciation	B×L	chr. 1:40	1.02	1:16,049,788.18,019,336	4.23	17.53			-0.33	
	*qKRN2*	Kernel row number	JP	chr. 2:18.8	2.02	2:4,139,916.4,808,238	5.77	13.23		5.45		0.69
		Kernel row number	L×L	chr2:27	2.02	2:4,335,580.5,766,846	7.76	22.99				0.87
	*qFas5*	Fasciation	L×L	chr5:114	5.07	5:210,666,787.211,006,289	4.09	21.70				-0.24
		Ovality	JP	chr5	5.07	5:216,124,262.218,020,826	4.51	11.41	1.88	2.64	-0.21	-0.34
	*qKRN5*	Kernel row number	L×L	chr5:154	5.07	5:217,164,610.218,092,335	3.83	10.01				-0.57
	*qKRN7.1*	Kernel row number	L×L	chr7:131	7.02	7:110,164,470.123,888,193	4.33	11.80				-0.62
	*qFas7*	Fasciation	JP	chr7:32.8	7.02	7:114,986,412.118,589,566	6.95	10.92	3.20	3.75	-0.23	-0.24
		Fasciation	B×L	chr7:44	7.02	7:114,986,412.118,512,477	4.58	19.41			-0.35	
		Disorder	JP	chr7:34.2	7.02	7:115,485,353.123,389,126	5.18	10.51	3.11	2.08	-0.43	-0.41
		Ovality	JP	chr7:39.4	7.02	7:125,598,407.125,842,182	4.65	11.48	*1.79*	2.85	*-0.19*	-0.36
	*qKRN7.2*	Kernel row number	JP	chr7:93.7	7.03	7:149,411,478.150,243,845	5.27	8.77		4.94		-0.65
	*qKRN8*	Kernel row number	L×L	chr8:34	8.02	8:18,827,357.20,248,512	6.29	17.88				-0.79
		Kernel row number	JP	chr8:23.5	8.02	8:19,522,583.20,248,512	4.71	8.30		4.53		-0.63
Tillering	*qTil1*	Tillering	JP	chr1:110.0	1.05	1:85,069,032.94,479,235	7.59	12.15	3.06	4.53	-0.40	-0.57
		Tillering	L×L	chr1:118.0	1.05	1:96,638,867.164,032,566	4.45	22.25				-0.85
	*qTil2*	Tillering	JP	chr2:7.3	2.01	2:2,067,198.3,242,152	7.05	13.08	6.42		0.58	
		Tillering	B×L	chr2:9	2.01	2:2,802,567.4,139,916	6.93	18.38			0.70	
	*qTil4*	Tillering	JP	chr4:115.9	4.04/05	4:30,890,749.37,691,500	6.70	10.66	3.19	3.51	-0.41	-0.51
	*qTil9*	Tillering	JP	chr9:67.5	9.03	9:92,749,841.97,243,143	6.19	9.50	3.40	2.79	-0.42	-0.45
Prolificacy	*qProl1 ^i^*	Prolificacy	L×L	chr1:2.0	1.01	1:6,272,408.7,074,707	6.07	5.58				0.34
	*qProl2*	Prolificacy	JP	chr2:139.2	2.06/7	2:187,831,696.191,179,806	6.34	13.97	4.52	*1.82*	0.13	*0.11*
	*qProl4 ^j^*	Prolificacy	L×L	chr4:371.1	4.05/4.08	4:148,677,638.181,859,161	6.22	5.74				0.34
	*qProl9*	Prolificacy	B×L	chr9:62	9.03	9:28,670,077.74,515,763	3.64	16.72			-0.21	

a ) Actual population (B×L, L×L or JP, with JP indicating the two populations jointly analyzed for QTL using the command ‘NAM’ in QTL Icimapping.

b ) QTL peak position in cM in the specific linkage map (B×L, L×L or JP, from this study).

c ) Peak LOD value from Composite Interval Mapping.

d ) PVE = Proportion of phenotypic variance explained.

e ) Peak LOD value of the single population B×L when analysed as JP. Sub-significant relevant LOD score are in Italics.

f ) Peak LOD value of the single population L×L when analysed as JP. Sub-significant relevant LOD score are in Italics.

g ) QTL additive effect express as 2a = (mean homozygous B73 – mean homozygous Lo1016). Additive values related with sub-significant LOD scores are in Italics.

h ) QTL additive effect express as 2a = (mean homozygous Lo964 – mean homozygous Lo1016). Additive values related with sub-significant LOD scores are in Italics.

i ) The position of *qProl1* was shifted to approx. 29 Mb based on Kruskal-Wallis test for ear prolificacy QTL (**Supplementary Table 3**).

j ) qProl4 was not detected based on Kruskal-Wallis test for ear prolificacy QTL (**Supplementary Table 3**).

### 3.3 QTL results

#### 3.3.1 Four ear-fasciation QTLs were identified and ear fasciation alleles were always contributed by Lo1016

**Figure 3:**
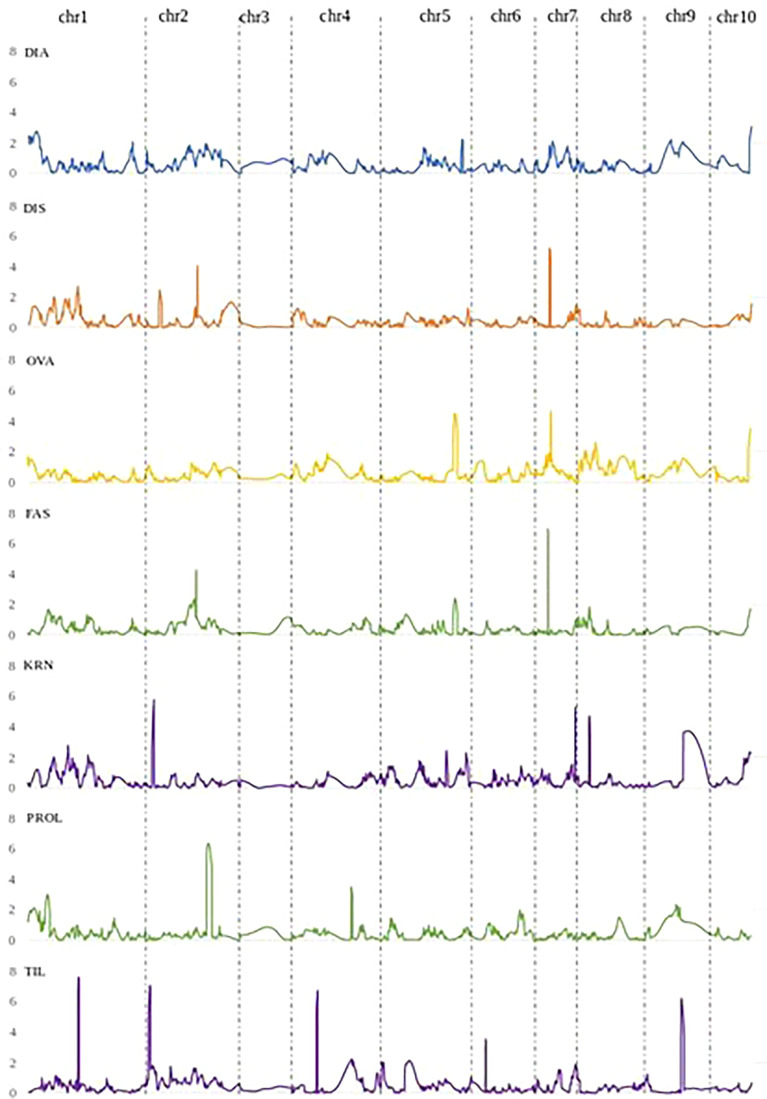
QTL LOD profiles obtained by the joint analysis of the two RIL populations B×L and L×L, for DIA (ear diameters rate), DIS (kernel row disorder), OVA (ear ovality), FAS (ear fasciation index), KRN (kernel row number), PROL (prolificacy), TIL (number of tillers).

In the following, QTLs for different ear fasciation-related traits (ear diameters rate, ear fasciation index, ear ovality, kernel disorder) will be considered as the same QTL whenever their supporting intervals overlap, considering QTL results from B×L, L×L and JP (**Figure 3** and **Supplementary Figures 5, 6**). Four QTL for ear fasciation traits were mapped (*qFas1.1* and *qFas1.2* on chr. 1; *qFas5* and *qFas7* on chr. 5 and 7, respectively. **Table 2**). *qFas1.1* and *qFas1.2* were detected for ear diameters rate and fasciation index, respectively, and mapped nearby on chr. 1 (bins 1.01/1.02) within two narrow supporting intervals of < 2 Mb, and both segregated within B×L only. *qFas5* was mapped on bin 5.07 for fasciation index and ear ovality and appeared to segregate mainly in L×L. *qFas7* was mapped on bin 7.02 and shown to affect ear fasciation index, ear ovality and kernel disorder in both B×L and L×L. Notably, for all *qFas* QTLs, the fasciation-increasing allele was provided by Lo1016, the parental line showing ear fasciation (**Figure 2**), as indicated by the direction of QTL effects (**Table 2**).

#### 3.3.2 KRN QTLs partially overlapped to ear fasciation QTLs

Five KRN QTLs were mapped. The QTL with the strongest effect, *qKRN2*, was mapped on bin 2.02 in a < 2 Mb-supporting interval and explained 13 or 23% (JP or L×L, respectively) of phenotypic variation with a genetic effect 2a = 0.69 or 0.87 kernel rows, in JP or L×L, respectively (‘+’ allele from Lo964). The QTL *qKRN5* on bin 5.07 controlled 10% of phenotypic variance and showed an effect of 2a = − 0.57 kernel rows (‘+’ allele contributed by Lo1016). Two KRN QTLs, *qKRN7.1* and *qKRN7.2*, mapped on bin 7.02 and 7.03, respectively, and had similar genetic effect (2a = −0.62 and −0.65 kernel rows, ‘+’ allele by Lo1016). The QTL *qKRN8* mapped on bin 8.02, with a genetic effect of 2a = −0.79 in L×L (‘+’ allele by Lo1016). Notably, all KRN QTLs segregated in L×L while none in B×L. Overall, *qKRN2* was the only KRN QTL with the ‘+’ allele contributed by Lo964, while Lo1016 contributed the ‘+’ allele at the other four KRN QTLs (**Table 2**). Notably, two out of five KRN QTLs overlapped with ear fasciation QTLs. Specifically, *qKRN5* overlapped with *qFas5* on bin 5.05 and *qKRN7.1* with *qFas7* on bin 7.02. At both chromosome regions, the ear fasciation-increasing allele (provided by Lo1016) also increased kernel row number, supporting the hypothesis of a functional association due to the presence of causative gene(s) affecting both ear fasciation and number of kernel rows, and in line with the observed positive correlation between the two traits (**Figure 2**).

#### 3.3.3 Ear prolificacy is under polygenic control in B×L and L×L populations independently from tillering

Four QTLs were mapped for ear prolificacy (*qProl1, qProl2*, *qProl4* and *qProl9.* **Table 2**). *qProl1* and *qProl4* were detected in L×L only, *qProl2* was mapped in JP and *qProl9* in B×L only. The highest PVE values were recorded for *qProl2* (14.0%) and *qProl9* (16.7%). At *qProl1*, *qProl2* and *qProl4*, the high ear prolificacy parent Lo964 contributed the ‘+’ QTL allele. Both *qProl1* and *qProl2* showed narrow physical supporting intervals (0.8 Mb and 3.3 Mb, respectively). Tillering variation was also shown to be under polygenic control with four QTLs. The two strongest QTLs in terms of genetic effect, *qTil1* and *qTil2*, mapped on bin 1.05 and 2.01, controlled 12-13% of phenotypic variance in JP with a genetic effect of 2a = 0.6 tillers per plant. Although the ‘+’ allele was contributed by Lo1016 for *qTil1, qTil4* and *qTil9*, in both B×L and L×L B73 contributed a positive tillering allele at *qTil2*. No overlap was found between prolificacy and tillering QTLs.

#### 3.3.4 Meristem genes *compact plant2* (*ct2*) and *ramosa1* (*ra1*) co-map with QTLs for ear fasciation *qFas1.2* and *qFas7*, and *barren inflorescence1* (*bif1*) co-maps with *qKRN8*

In order to search for candidate genes of ear fasciation QTLs identified in this study, we extracted all gene models included in the QTL supporting intervals present in B73 v4 (www.maizegdb.org) along with gene expression information in meristem and ear primordium. Alongside, a list of 42 genes involved in development and/or proliferation of ear meristem was collected by screening the scientific literature (**Supplementary Table 5**); a sublist of genes comapping with QTLs in our study is provided in **Supplementary Table 6**. For instance, at *qFas1.2* (chr. 1, 16.0-18.0 Mb), *compact plant2* (*ct2.* Bommert et al., 2013a), and *big grain1 homolog1* (*bgh1.* Simmons et al., 2020) at chr. 1, 16.7 Mb, were identified as candidate genes. The well-known *ra1* (Vollbrecht et al., 2005; Dempewolf, 2010) on chr. 7, 113.6 Mb, is included in the supporting interval of *qFas7*/*qKRN7.1* (chr. 7, 110.2-123.9 Mb). Additionally, at *qKRN8* (chr. 8, 18.8–20.2 Mb), *barren inflorescence1* (*bif1.* Barazesh and McSteen, 2008), chr. 8, 18.9 Mb was identified as candidate gene for KRN. As far as tillering is concerned, two candidate genes, namely *crr1* (*cytokinin response regulator1*, gene model Zm00001d001865) and *arftf3* (*ARF-transcription factor 3*, gene model Zm00001d001879) were identified within the supporting interval of *qTil2*, on chr. 2.

#### 3.3.4 Investigation of nucleotide and amino acid sequence variation at candidate genes for ear fasciation, number of kernel rows QTLs and tillering

The nucleotide sequences of candidate genes listed in **Supplementary Table 6** were recovered for the three parental lines based on the reference genome sequence (B73 v4 from www.maizegdb.org) or based on the *de novo* whole genome shotgun sequences obtained in this work (Lo964 and Lo1016), and compared in order to identify functional variants. Specifically, variants were searched for *ra1* (candidate at *qKRN7.1*/*qFas7*), *ct2* and *bgh1* (candidates at *qFas1.2*) and *crr1* (candidate for *qTil2*. **Supplementary Figures 7-11**). However, in all these cases, no nucleotide difference was observed between the parental alleles. This result does not rule out *ra1*, *ct2*/*bgh1* and *crr1* as possible candidate genes for their QTLs, instead, it suggests that the candidate genes could act on ear fasciation by gene expression changes.

## 4 Discussion

We phenotyped ear fasciation using four approaches, namely collecting the rate between the minor and the major cob diameters, ear ovality or flatness, kernel row disorder index and an ear fasciation index. Thus, our phenotyping approaches covered well the different ways ear fasciation manifests, namely cob flatness and kernel disorder as shown previously (Mendes-Moreira et al., 2015; Kim et al., 2022). Confirming other authors’ observations, cob ovality/flatness and kernel row disorder correlated, and correlated also with ear fasciation index. Additionally, cob ovality, kernel disorder and ear fasciation index QTLs showed a sizeable level of overlap. At the same time, both the imperfect correlation between such traits (e.g., *r* = 0.67 between ear ovality/flatness and kernel row disorder) and the presence of QTLs affecting only one out of four ear-fasciation-related traits (e.g., *qFas1.1*, controlling ear diameters rate) confirmed that ear fasciation is a genetically and physiologically complex polygenic trait and that at least some genes can possibly affect kernel disorder without affecting cob ovality/flatness, or vice versa. The presence of QTLs specific for single components of ear fasciation was already reported (Mendes-Moreira et al., 2015).

Confirming earlier observations, phenotypic variation for ear fasciation and KRN were found associated in our study, both in terms of positive correlation and QTLs overlap. Specifically, two out of four ear fasciation QTLs overlapped with two out of five KRN QTLs. Furthermore, at the overlapping loci, namely *qFas5-qKRN5* and *qFas7-qKRN7.1*, the direction of genetic effects agreed (i.e., ‘+’ alleles increased both ear fasciation index and the number of kernel rows), as previously hypothesized or shown. Notably, at both *qFas5-qKRN5* and *qFas7-qKRN7.1* the fasciation/KRN-increasing allele was provided by Lo1016, and the effect of the Lo1016 alleles at the two QTLs was detected in both B×L and L×L. In other words, at both *qFas5-qKRN5* and *qFas7-qKRN7.1*, Lo1016 carries alleles increasing both ear fasciation index and kernel row number, and their positive effect on ear fasciation were detected across genetic backgrounds. However, the KRN effect was detected in the L×L background only, likely because the KRN mean value in the L×L genetic background was lower than that in B×L (16.0 and 17.4 kernel rows per ear, respectively; **Table 1**). Indeed, in a high KRN-context such as the B×L genetic background, any KRN-increasing allele such as the ones from Lo1016 would likely contribute marginally to KRN. In L×L, the genetic effect at both *qFas5-qKRN5* and *qFas7-qKRN7.1* QTLs was estimated to be 2a = approx. 0.6 rows per locus (|0.57| at *qKRN5* and |0.62| at *qKRN7.1.* **Table 2**), equivalent to approx. 4% (0.6/16.5 rows per ear) of the average trait value in these populations. Homozygous Lo1016 allele substitutions at both loci are therefore expected to add approximately one row per ear, therefore contributing approximately 6% (1/16.5 rows) of grain yield. Although this estimate should be considered with caution, the combined effect of the two QTLs on kernel per ear seems important and worthy to consider in plant breeding programs when based on marker- or genomics-assisted selection.

**Figure 4:**
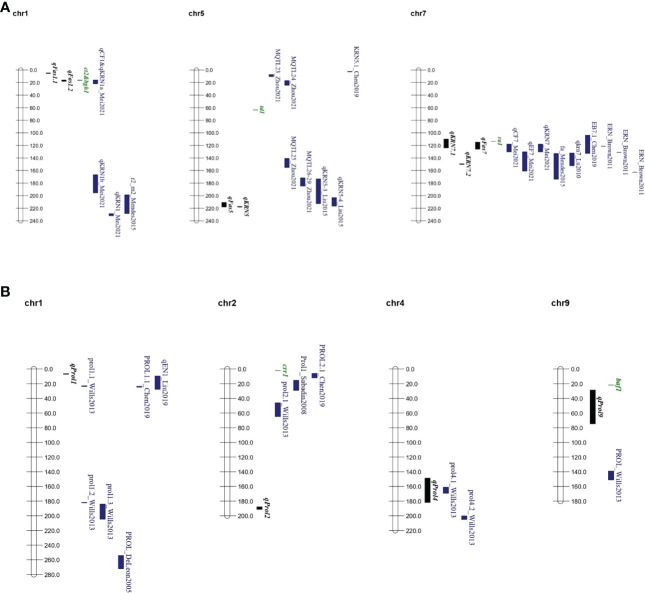
QTL and main candidate genes consensus maps. **(A)** QTL consensus map for ear fasciation including QTL from literature and this study. **(B)** QTL consensus map for ear prolificacy, including QTL from literature and from this study. Chromosome bars and numbers represent physical distances in Mb. QTL positions are represented following physical positions reported in **Supplementary Tables 7, 8**. In black, QTL from this study; in green, tentative candidate genes; in blue, QTL from other studies. Full QTL information from other studies is provided in **Supplementary Table 7**.

Our QTL consensus map (**Figure 4A**, **Supplementary Table 7**) supported the pleiotropic connection between ear fasciation and KRN. For example, QTLs for cob ovality and KRN (*qCF1* and *qKRN1a*, respectively) by Mei et al. (2021) on chr. 1 overlapped with *qFas1.2* mapped in our study. Liu et al. (2015) found a QTL (*qKRN5-4*) between *umc1971* and *umc1071* on chr. 5 affecting kernel row number, and mapping nearby our *qFAS5*. Finally, the chr. 7 region corresponding to our *qFas7-qKRN7.1* appears as an ear fasciation/KRN QTL hot spot. QTLs mapped in this region included cob and ear flatness *qCF7* and *qEF7* by Mei et al. (2021), ear fasciation *fa_c1* by Mendes-Moreira et al. (2015), several ear row number QTLs within the B73 NAM population by Brown et al. (2011), *qkrn7* by Lu et al. (2011), *KRN7.1* by Chen et al. (2019), *qKRN7* by Mei et al. (2021).

We also detected QTLs affecting the number of kernel rows independently from ear fasciation on chr. 2 and chr. 8 (*qKRN2* and *qKRN8*, respectively. **Table 2**) with (+) alleles dispersed between parental lines (from Lo964 and Lo1016, respectively). Many independent QTL mapping studies for number of kernel rows have already been carried out and a comprehensive review is beyond the scope of this study. However, it should be noted that a major KRN QTL mapping on chr. 2, just 10 Mb away from *qKRN2* was cloned and shown to encode for a member of the highly duplicated *WD40* gene and protein family (Chen et al., 2022), which affects diverse cellular functions like signal transduction, cell cycle control, intracellular transport, chromatin remodelling, cytoskeletal organization and others. The authors reported that the *WD40* allele increasing the number of kernel rows enhanced the inflorescence meristem size, likely providing additional space for initiation of spikelet pair meristems and hence a higher number of kernel rows (Chen et al., 2022). Given the close proximity (ca. 10 Mb) between *WD40* and the QTL *qKRN2* reported herein in the subtelomeric region of chr. 2, it will certainly be worth checking the actual identity between the two loci.

By comparing QTL supporting intervals from our and other studies with the genomic positions of inflorescence-related genes we shortlisted candidate genes possibly involved in controlling ear fasciation QTLs (**Figure 4A**, **Supplementary Tables 5, 6**). The maize historical tassel and ear mutant *ra1*, encoding a zinc-finger transcriptional factor and producing ear and tassel with increased branches (Vollbrecht et al., 2005; Kim et al., 2022) maps only 0.5 Mb away from the QTL cluster region including *qFas7*, and within the QTL supporting interval of *qKRN7.1*. Comparison of genomic sequences between our three parental lines showed lack of nucleotide sequence variation at *ra1* (**Supplementary Figure 8**), in line with former observations which showed *ra1* as very poor of sequence diversity in maize (Sigmon and Vollbrecht, 2010). However, as shown in other studies (Salvi et al., 2007; Li et al., 2012; Salvi and Tuberosa, 2015), QTLs are often due to gene expression level variation rather than variation of coding sequences, therefore quantification of the expression of *ra1* in the ear primordium of Lo1016 and Lo964 will enable to test *ra1* involvement in ear fasciation driven by *qFas7*. The genes *ct2* and *bgh1* were identified as candidate genes for *qFas1.2* (**Supplementary Table 6**) based on former observations that maize lines carrying mutations at *ct2* produced fasciated ears (Bommert et al., 2013a), and that the overexpression of *bgh1* resulted in increased ear kernel row number (Zhang et al., 2022). Five common native *bgh1* alleles exhibited little structural and expression variation compared to the large increased expression conferred by these ectopic alleles (Simmons et al., 2020). In line with this observation, genomic sequence comparison between B73 and Lo1016 (*qFas1.2* was detected in B×L only; **Table 2**) showed no difference in coding sequences.

Our study addressed single-node ear prolificacy (Wang et al., 2021), a trait hardly investigated across maize genetics and we identified four major QTLs. An overlap was observed between our *qProl4* and *prol4.1* by Wills et al. (2013) in a maize × teosinte cross (**Figure 4B**, **Supplementary Table 8**), although no association with known genes inside this interval was established. Additionally, *qProl1* mapped in the proximity of three ear prolificacy QTLs reported in other studies (Wills et al., 2013; Chen et al., 2019; Liu et al., 2019; **Figure 4B**, **Supplementary Table 8**), The gene *grassy tillers1* (*gt1*) known to be involved in ear prolificacy (Wills et al., 2013) maps just ~16 Mb away from *qProl1* (**Table 2**; **Supplementary Table 5**). Finally, *barren stalk fastigiate1* (*baf1*), a known gene that when mutated produces barred shoots with no ear (Zhou et al., 2021) maps very close (~7 Mb) to the north border of *qProl9* (**Figure 4B**). Thus, given the vagaries of QTL mapping, both *gt1* and *baf1* cannot be excluded as possible causative genes for their corresponding QTLs.

Among the four QTLs controlling the number of tillers per plant, three (*qTil1*, -*4* and -*9*) had the tillers-increasing allele provided by Lo1016, in line with the phenotype of parental lines (Lo1016 is the only parent showing tillering when grown in the field in standard conditions). However, *qTil2* had the tiller-increasing allele contributed by B73 that develops hardly no tillers in our field conditions (**Table 1**), which suggests that at least some level of epistasis occurs between tillering loci in our genetic materials, in line with former observations of epistasis for domestication traits, including tillering (Stitzer and Ross-Ibarra, 2018). Finally, it should be noted that while shoot branching producing ears and tillering share obvious developmental similarities (e.g., both branching types originate from axillary buds at stem nodes), ear prolificacy and tillering did not correlate and did not show QTL overlap. The most likely explanation lies in the genetic architecture of the two traits in the lines tested in this study, i.e., no strong pleiotropic gene segregated. Another factor, partially connected with this, is that the parental line contributing ear prolificacy (Lo964) showed virtually no tillering, and the parental line contributing high tillering (Lo1016) showed no ear prolificacy, suggesting that each parental line possibly contributed relatively strong alleles at genes acting only on one of the two traits.

## 5 Conclusions

This study identified solid positive correlation between ear fasciation and KRN in an elite genetic background, and provided evidence that the correlation was at least partially due to pleiotropic genes at ear fasciation QTLs on chr. 5 and chr. 7 and affecting KRN. The fasciation effects and the correlated effect on KRN were confirmed across genetic backgrounds, making these QTLs an interesting source of yield-positive alleles. While candidate genes were identified at major QTLs, including the correspondence between *qFas7-qKRN7.1* and *ra1* on chr. 7, further work is required for candidate genes validation.

Analysis for ear prolificacy at a single node enabled us to identify four QTLs, of which one (on chr. 4) perfectly overlapped with an ear prolificacy QTL formerly identified in a maize × teosinte cross. Quite unexpectedly, we did not find correlation or QTL overlaps between ear prolificacy and tillering, although the two traits share obvious developmental basis.

Overall, our study provides clear entry points for the molecular dissection of important yield component traits, which should help both developing molecular markers for marker-assisted selection to be deployed in breeding programs and starting the procedures leading to cloning the genes underpinning the QTLs described and eventually their manipulation by engineering or editing.

## Data availability statement

The raw data supporting the conclusions of this article will be made available by the authors, without undue reservation.

## Author contributions

SS and RT conceived the project and acquired funding; KL, AT, SG, SR, CU and SS designed and curated field trials, collected phenotypic and genotypic data and analyzed the data; KL, RT and SS wrote the first manuscript draft. All authors contributed to the article and approved the submitted version.
