# Supplementary material for: QTL mapping identifies novel major loci for kernel row number-associated ear fasciation, ear prolificacy and tillering in maize (Zea mays L.)
Source: Front Plant Sci. 2023 Jan 10;13:1017983. doi: 10.3389/fpls.2022.1017983 (PMC9871824; doi:10.3389/fpls.2022.1017983)
Supplement: Supplementary file 1 [file DataSheet_1.zip › Supplementary Material Figures.pdf]

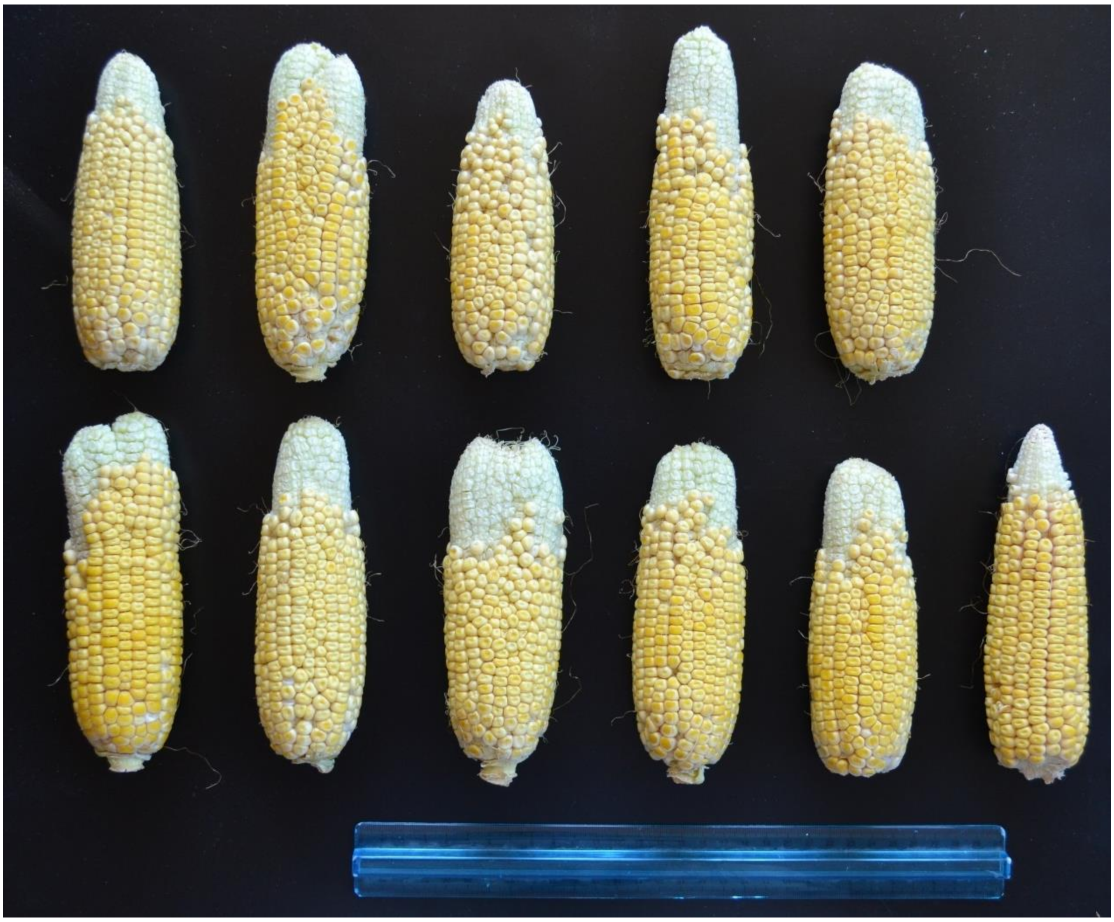

Supplementary figure 1. Lo1016 top ears collected from ten contiguous plants grown in the same plot, at late waxy maturation stage. On the right, an ear from a randomly chosen Lo964 plant from a nearby plot is shown. Ruler length is 30 cm.

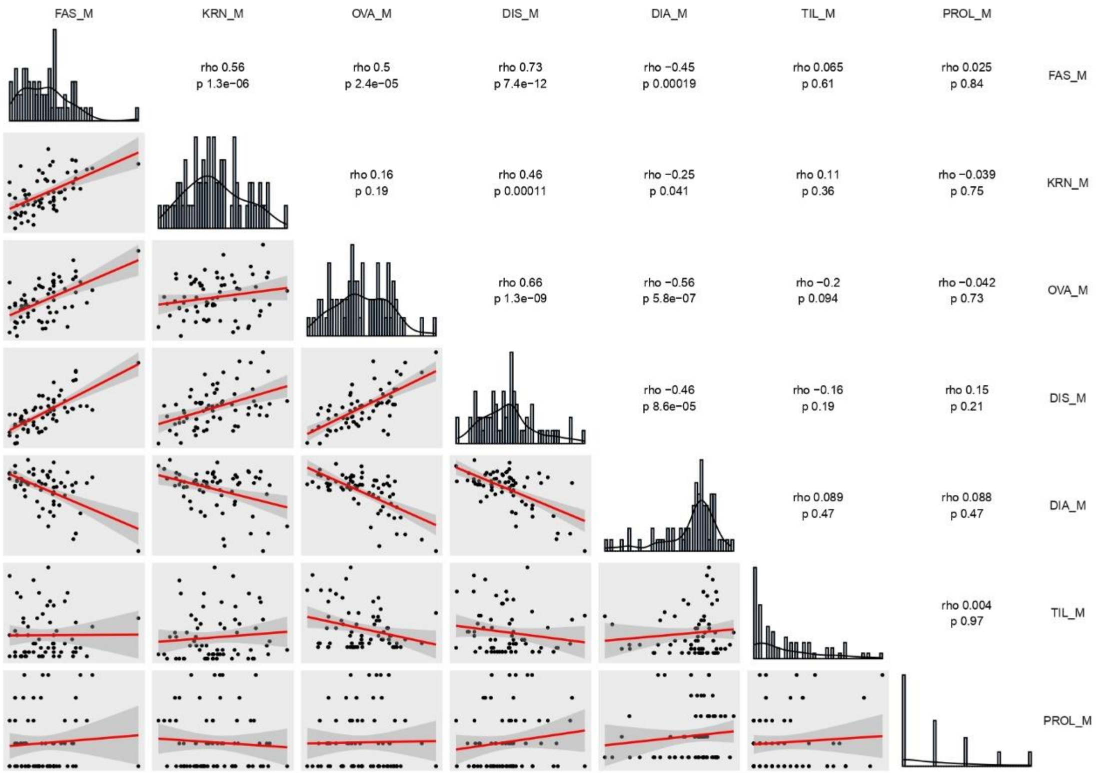

Supplementary Figure 2. Distribution frequency histograms of, and correlation among all traits estimated on the B×L. The upper right part presents all correlation indexes (*rho*) and corresponding significant levels (*p*). The lower left part presents scatter plots and fitter curve (the red line inside) between two traits. The diagonal shows histogram charts of each trait. DIA (ear diameters rate), DIS (kernel row disorder), OVA (ear ovality), FAS (ear fasciation index), KRN (kernel row number), PROL (prolificacy), TIL (number of tillers), M, mean value.

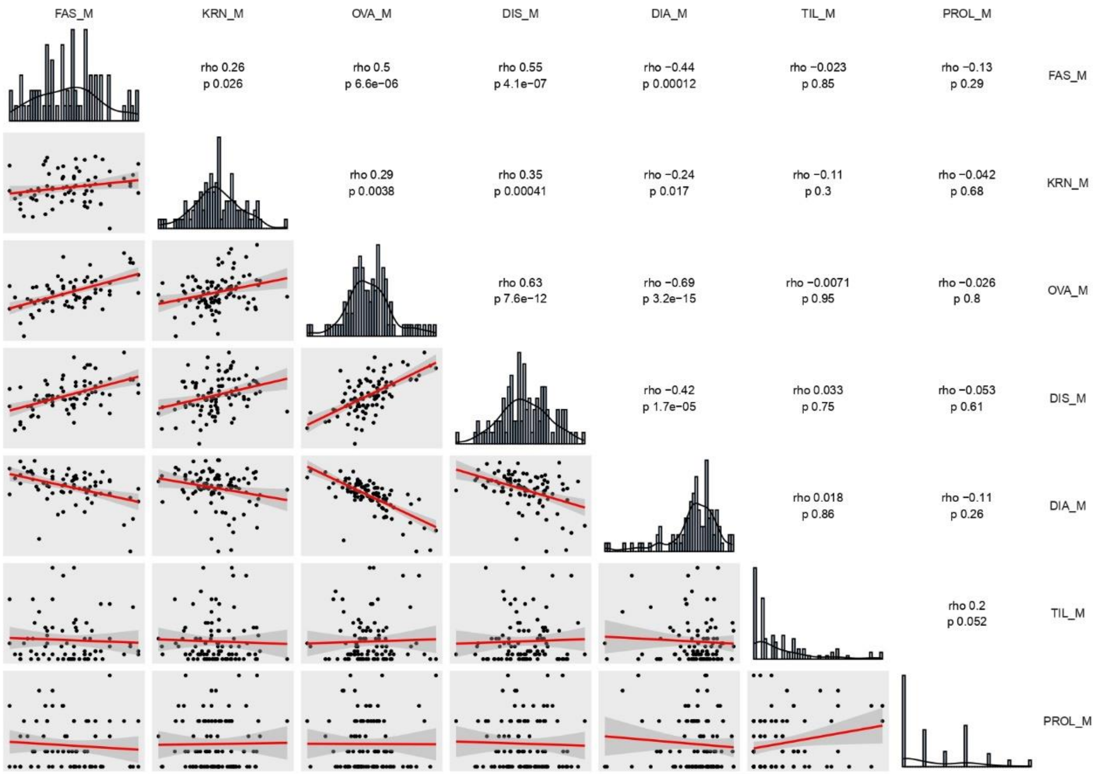

Supplementary Figure 3. Distribution frequency histograms of, and correlation among all traits estimated on the L×L. The upper right part presents all correlation indexes (*rho*) and corresponding significant levels (*p*). The lower left part presents scatter plots and fitter curve (the red line inside) between two traits. The diagonal shows histogram charts of each trait. DIA (ear diameters rate), DIS (kernel row disorder), OVA (ear ovality), FAS (ear fasciation index), KRN (kernel row number), PROL (prolificacy), TIL (number of tillers), M, mean value.

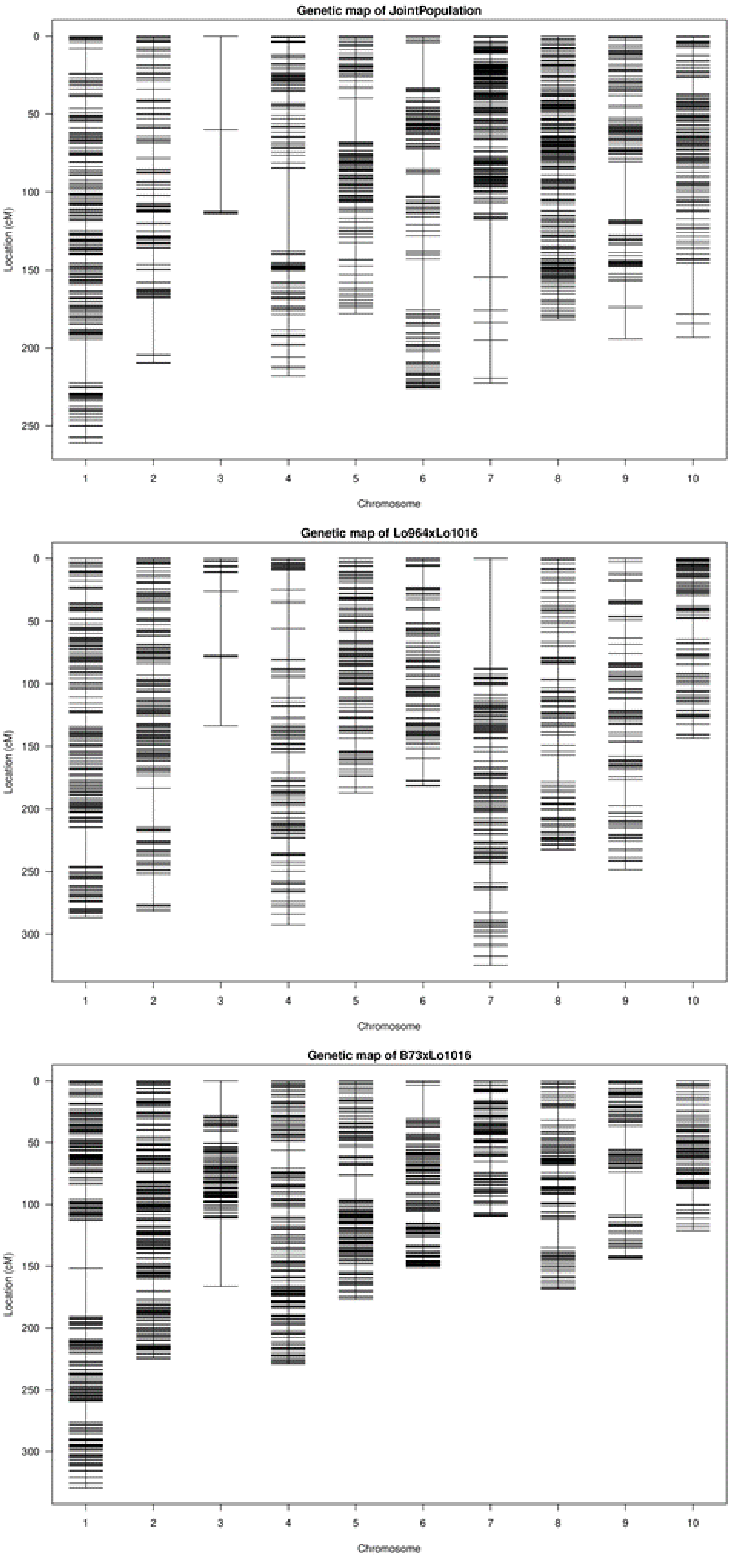

Supplementary Figure 4. Linkage map plots. From top to bottom: Joint Population map, Lo964 × Lo1016, B73 × Lo1016.

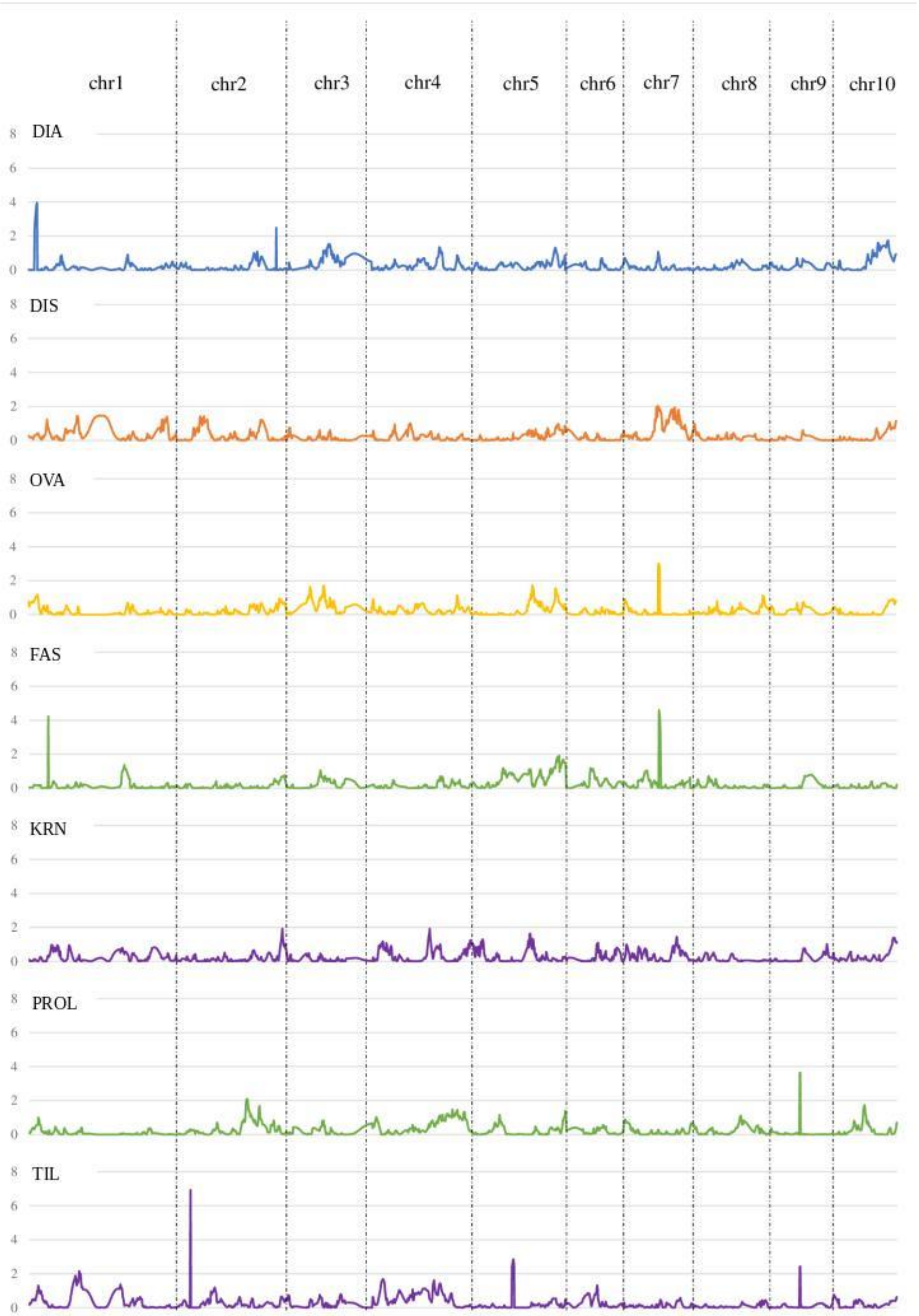

Supplementary Figure 5. QTL LOD profiles obtained in the RIL population B73 × L01016, for DIA (ear diameters rate), DIS (kernel row disorder), OVA (ear ovality), FAS (ear fasciation index), KRN (kernel row number), PROL (prolificacy), TIL (number of tillers).

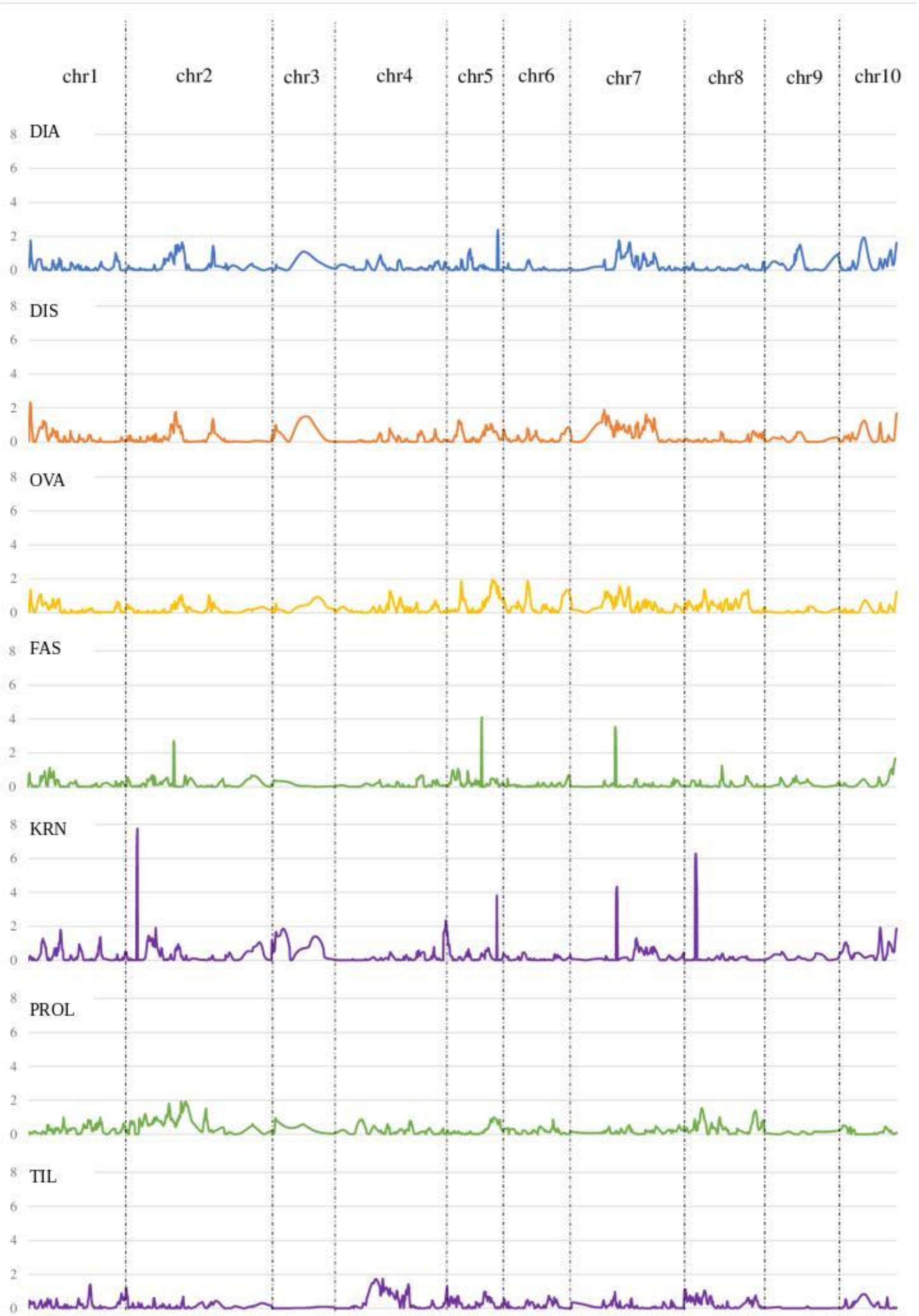

Supplementary Figure 6. QTL LOD profiles obtained in the RIL population Lo964 × Lo1016, for DIA (ear diameters rate), DIS (kernel row disorder), OVA (ear ovality), FAS (ear fasciation index), KRN (kernel row number), PROL (prolificacy), TIL (number of tillers).

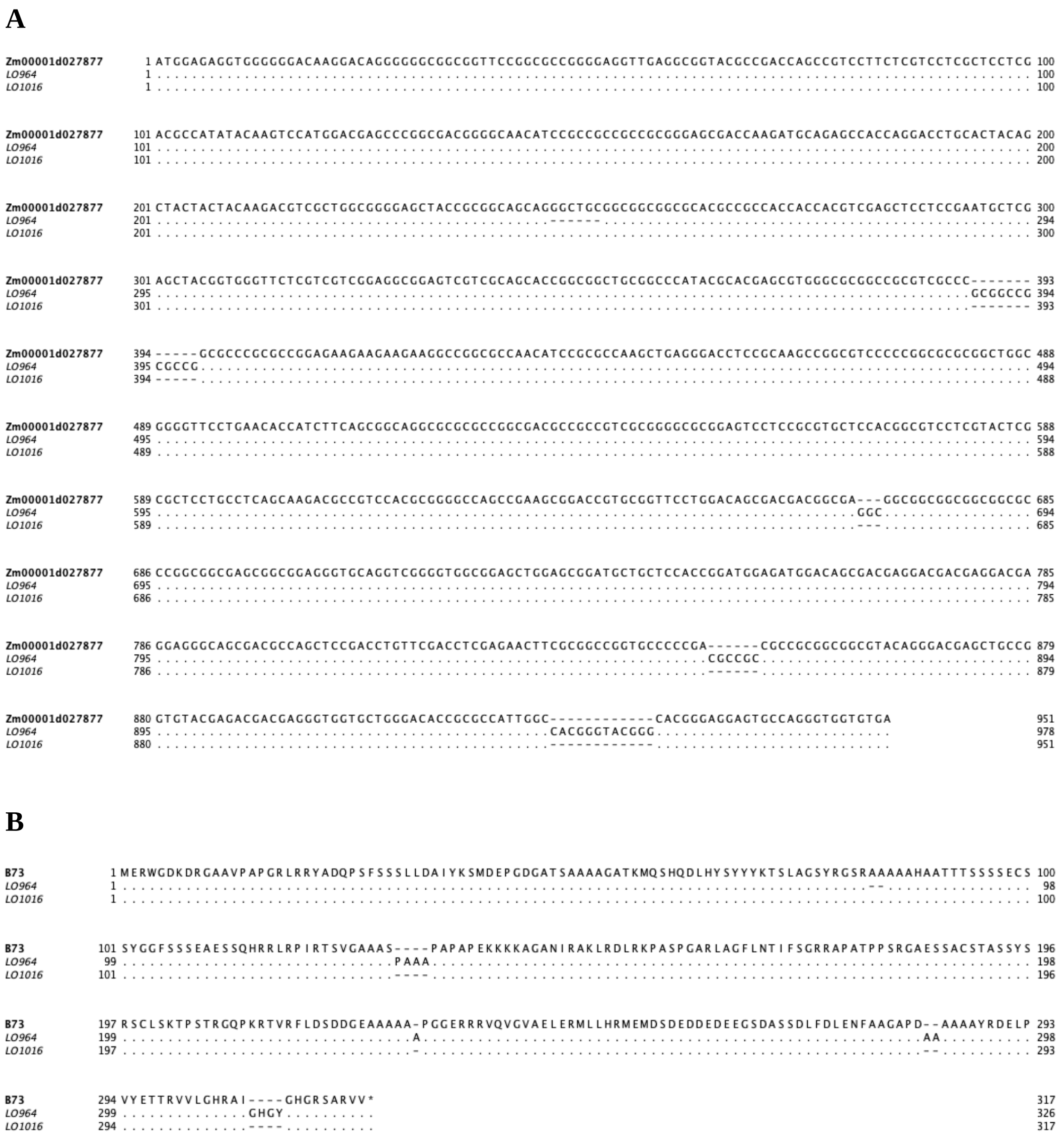

Supplementary Figure 7. Multi-alignment of *Bgh1* alleles. Alignment of nucleotides of the coding sequence A) and of the corresponding amino acids B) from Lo964 and Lo1016 to the reference sequence B73 version 4.

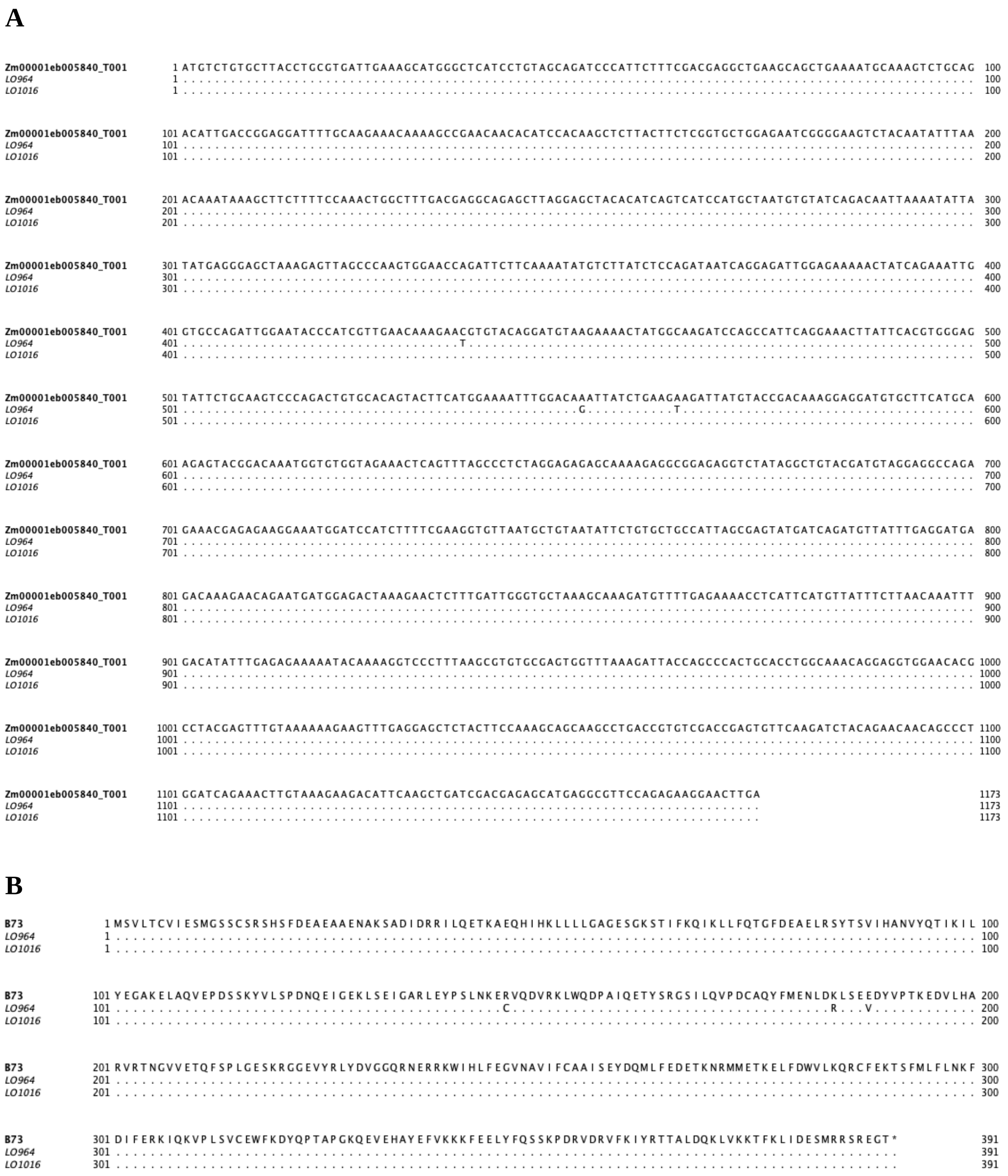

Supplementary Figure 8. Multi-alignment of *ct2* alleles. Alignment of nucleotides of the coding sequence A) and of the corresponding amino acids B) from Lo964 and Lo1016 to the reference sequence B73 version 4.

A

```
Zm00001eb312340_T001   1 ATGGAGGGAGAAGATGACGGCGCCCAAATGAAACTGCAGCAACAACAACAGTCGCCTTGCAGTGACAACTTGAGCTTGTCCGCCGCCTCCTCATGGCTGC 100
LO964                  1 .................................................................................................... 100
LO1016                 1 .................................................................................................... 100

Zm00001eb312340_T001 101 CGCCACAGGTAAGGTCGTCGTCGTCGTCGTCGTCGTACACCTGCGGGTATTGCAAGAAGGAGTTCAGATCAGCACAAGGGCTGGGAGGCCACATGAACAT 200
LO964                101 .................................................................................................... 200
LO1016               101 .................................................................................................... 200

Zm00001eb312340_T001 201 CCACAGGCTGGACAGGGCCAGACTGATCCACCAACAGTACACTTCACACCGTATTGCTGCTCCCCATCCAAACCCTAATCCTAGTTGCACATCAGTTCTT 300
LO964                201 .................................................................................................... 300
LO1016               201 .................................................................................................... 300

Zm00001eb312340_T001 301 GACCTTGAGCTCAGCTTGTCGTCGCTGCTAGCGCATGGTGCTGCCAGCAGCGACGGAGGCTTGTCTGTTCCAGTGGCAAAGCTGGCGGGCAACCGTTTCT 400
LO964                301 .................................................................................................... 400
LO1016               301 .................................................................................................... 400

Zm00001eb312340_T001 401 CCTCCGCATCGCTCCCCACGACCAAGGACGTCGAGGGGAAGAACTTAGAGTTGAGGATAGGAGCGTGCAGTCATGGCGATGGCGCGGAAGAGCGTCTGGA 500
LO964                401 .................................................................................................... 500
LO1016               401 .................................................................................................... 500

Zm00001eb312340_T001 501 TCTTCAGCTTAGACTGGGCTACTACTGA 528
LO964                501 ............................ 528
LO1016               501 ............................ 528
```

B

```
B73      1 MEGEDDGAQMKLQQQQQSPCSDNLSLSAASSWLPPQVRSSSSSSSYTCGYCKKEFRSAQGLGGHMNIHRLDRARLIHQQYTSHRIAAPHPNPNPSCTSVL 100
LO964    1 .................................................................................................... 100
LO1016   1 .................................................................................................... 100

B73    101 DLELSLSSLLAHGAASSDGGLSVPVAKLAGNRFSSASLPTTKDVEGKNLELRIGACSHGDGAEERLDLQLRLGYY* 176
LO964  101 ............................................................................ 176
LO1016 101 ............................................................................ 176
```

Supplementary Figure 9. Multi-alignment of *RA1* alleles. Alignment of nucleotides of the coding sequence A) and of the corresponding amino acids B) from LO964 and LO1016 to the reference sequence B73 version 4.

A

```
Zm00001eb336930_T004   1 ATGTCGGTGGATACGGAGCGGAGCTCCACCGAGTCGTCCGCGGCCTCCGGGCTCGGCTACGAGGACACCGCGCTCGCCCTCACCCTCCGCCTCCCGGGCT 100
LO964                  1 .................................................................................................... 100
LO1016                 1 .................................................................................................... 100

Zm00001eb336930_T004 101 CCGACCCCGGCCGTTCCTCCCCGCTCGCCGCGCCCTCCGACGCCGCGCCGTCTCCCAAGACGCGGGTGGTGGGGTGGCCGCCGGTGAGGTCGTACCGCAA 200
LO964                101 .................................................................................................... 200
LO1016               101 .................................................................................................... 200

Zm00001eb336930_T004 201 GAACGCGCTCGCCGACTCCAGCAAGGCCAACCGGTCAGCCAGTTTCGTGAAGGTGGCCGTCGACGGCGCGGCCTACCTGCGGAAGGTGGACCTCCAGGCG 300
LO964                201 .................................................................................................... 300
LO1016               201 .................................................................................................... 300

Zm00001eb336930_T004 301 TACGGCGGCTACGACCAGCTCCTCCGCGCGCTCCAGGACAAGTTCTTCTCCCACTTCACCATCAGGAAGTTCGCCGACGACGAGAGGAAGCTGGTGGACG 400
LO964                301 .................................................................................................... 400
LO1016               301 .................................................................................................... 400

Zm00001eb336930_T004 401 CGGTGAACGGGACGGAGTACGTGCCCACGTACGAGGACAAGGATGGCGACTGGATGCTCGTCGGCGACGTCCCCTGGAAGATGTTCGTGGAGACCTGCCG 500
LO964                401 .................................................................................................... 500
LO1016               401 .................................................................................................... 500

Zm00001eb336930_T004 501 GCGCCTTCGCCTGATGAAAGGTTCAGAGGCCGTTAACTTGGCACCAAGAGCCGCCCGATGA 561
LO964                501 ............................................................. 561
LO1016               501 ............................................................. 561
```

B

```
B73      1 MSVDTERSSTESSAASGLGYEDTALALTLRLPGSDPGRSSPLAAPSDAAPSPKTRVVGWPPVRSYRKNALADSSKANRSASFVKVAVDGAAYLRKVDLQA 100
LO964    1 .................................................................................................... 100
LO1016   1 .................................................................................................... 100

B73    101 YGGYDQLLRALQDKFFSHFTIRKFADDERKLVDAVNGTEYVPTYEDKDGDWMLVGDVPWKMFVETCRRLRLMKGSEAVNLAPRAAR* 187
LO964  101 ....................................................................................... 187
LO1016 101 ....................................................................................... 187
```

Supplementary Figure 10. Multialignment of *bif1* alleles. Alignment of nucleotides of the coding sequence A) and of the corresponding amino acids B) from LO964 and LO1016 to the reference sequence B73 version 4.

A

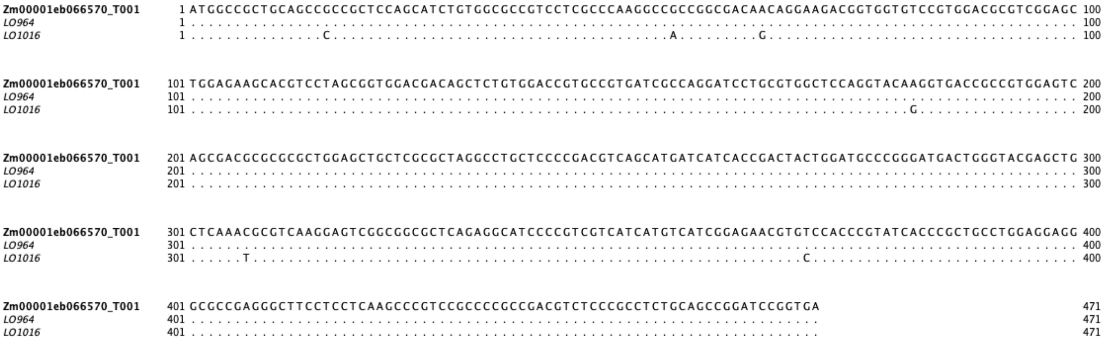

B

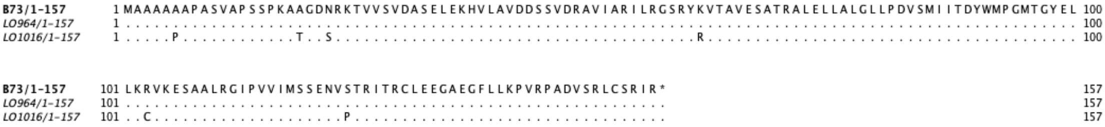

Supplementary Figure 11. Multialignment of *crr1* alleles. Alignment of nucleotides of the coding sequence A) and of the corresponding amino acids B) from LO964 and LO1016 to the reference sequence B73 version 4.
